# Molecular characterization of a *Trichinella spiralis* serine proteinase

**DOI:** 10.1186/s13567-020-00847-0

**Published:** 2020-09-25

**Authors:** Xin Yue, Xiang Yuan Sun, Fang Liu, Chen Xi Hu, Ying Bai, Qi Da Yang, Ruo Dan Liu, Xi Zhang, Jing Cui, Zhong Quan Wang

**Affiliations:** Department of Parasitology, Medical College, Zhengzhou University, Zhengzhou, 450052 China

**Keywords:** *Trichinella spiralis*, serine proteinase, invasion, intestinal epithelial cells (IECs), immunogenicity, ADCC

## Abstract

The aim of this study was to investigate the biological characteristics and functions of a *Trichinella spiralis* serine proteinase (TsSerp) during larval invasion and development in the host. The full-length TsSerp cDNA sequence was cloned and expressed in *Escherichia coli* BL21. The results of RT-PCR, IFA and western blotting analyses showed that TsSerp was a secretory protein that was highly expressed at the *T. spiralis* intestinal infective larva and muscle larva stages and primarily located at the cuticle, stichosome and intrauterine embryos of the parasite. rTsSerp promoted the larval invasion of intestinal epithelial cells (IECs) and the enteric mucosa, whereas an anti-rTsSerp antibody impeded larval invasion; the promotion and obstruction roles were dose-dependently related to rTsSerp and the anti-rTsSerp antibodies, respectively. Vaccination of mice with rTsSerp elicited a remarkable humoral immune response (high levels of serum IgG, IgG1/IgG2a, IgE and IgM), and it also triggered both systemic (spleen) and local intestinal mucosal mesenteric lymph node (MLN) cellular immune responses, as demonstrated by a significant elevation in Th1 cytokines (IFN-γ) and Th2 cytokines (IL-4) after the spleen and MLN cells from vaccinated mice were stimulated with rTsSerp. Anti-TsSerp antibodies participated in the killing and destruction of newborn larvae via ADCC. The mice vaccinated with rTsSerp exhibited a 48.7% reduction in intestinal adult worms and a 52.5% reduction in muscle larvae. These results indicated that TsSerp participates in *T. spiralis* invasion and development in the host and might be considered a potential candidate target antigen to develop oral polyvalent preventive vaccines against *Trichinella* infection.

## Introduction

The genus *Trichinella*, including 10 species and 3 genotypes, is distributed in more than 150 domestic and wild mammals, and some species of the genus may cause the meat-borne zoonosis trichinellosis [1]. Humans acquire trichinellosis by consuming raw or semi-raw meat contaminated with *Trichinella* infective larvae. In China, endemic areas of human trichinellosis are mainly located in the southwestern region, but *Trichinella* infection in humans and animals was found in 33 of 34 provinces [2]. From 2004 to 2009, 12 trichinellosis outbreaks due to infected pork were reported [3]. Domestic pork is still the primary infectious source of *Trichinella* infection in developing countries [4–6]. Because *Trichinella* is a zoonotic parasite and has a broad distribution of natural hosts in the world, it is difficult to eradicate *Trichinella* infection in animals. *Trichinella* infection poses a tremendous hazard to public health and animal food safety [7]. The development of preventive anti-*Trichinella* vaccines might be an effective strategy to control and eliminate *Trichinella* infection in food animals. Therefore, it is necessary to identify and characterize novel target antigens for use as vaccine molecules against *Trichinella* infection.

Following ingestion, encapsulated muscle larvae (MLs) of *T. spiralis* are liberated from their collagen capsules through gastric fluid digestion and then develop into intestinal infective larvae (IILs) after being exposed to enteric contents or bile [8, 9]. The IILs invade the enteric epithelium and grow to adulthood worms (AWs) after moulting 4 times. After copulation, pregnant female adults deposit newborn larvae (NBLs), which enter the blood, penetrate into the skeletal muscles and encapsulate to accomplish their life cycle [10]. In the process of the *T. spiralis* lifecycle, the excretory/secretory (ES) antigens of MLs and IILs are first exposed to host intestinal epithelium cells (IECs), and they might exert an important role in larval invasion and trigger the early immune response [11]. By using proteomics/immunoproteomics techniques, some serine proteases have been found among the ES or surface proteins of *T. spiralis* worms [12–14]. Serine proteases are a family of proteolytic enzymes that have multiple biological roles during parasite infection, and they are involved in worm invasion, migration and proteolysis of the host’s various tissues [15, 16]. Serine proteases might participate in *T. spiralis* larval invasion of IECs [17], and they might be important antigenic molecular targets for anti-*Trichinella* vaccines [18, 19].

Previous studies have shown that several kinds of serine proteases participate in the invasion of IECs by *T. spiralis* infective larvae. However, vaccination of mice with individual recombinant serine proteases produced only partial immune protection against *T. spiralis* larval challenge infection [20–22]. Therefore, it is necessary to characterize other newly discovered *T. spiralis* serine proteases and investigate their immunogenicity. In previous studies, a *T. spiralis* serine proteinase (TsSerp; GenBank: AY028974.1) was identified among major ES proteins of *T. spiralis* MLs by immunoproteomics [23]; it was mainly located in MLs at 30 days post-infection (dpi) [24].

The aim of this study was to further investigate the biological characteristics and functions of TsSerp during *T. spiralis* infection and to assess its immunogenicity.

## Materials and methods

### Parasites, animals and antigens

The parasite *T. spiralis* (ISS534) was acquired from a domestic pig in central China [25] and maintained in our laboratory by serial passaging in BALB/c mice. Four- to six-week-old female BALB/c mice were purchased from Henan Experimental Animal Center. The MLs were collected through artificial digestion of infected murine carcasses at 40 dpi [26]. The IILs and AWs were recovered from the small intestine of infected mice at 6 hpi and 3 and 5 dpi [27]. Adult females at 5 dpi were cultured in RPMI-1640 with 10% foetal bovine serum (FBS; Gibco) at 37 °C in 5% CO_2_ for 24 h, and NBLs were harvested as previously described [28, 29]. The somatic crude proteins of various stage *T. spiralis* worms (MLs, IILs, 3 dpi AWs and NBLs) and ML ES proteins were prepared as reported [30]. In brief, the worms were first homogenized using a high-speed tissue grinder (KZ-II Servicebio), and worm fragments were further homogenized by ultrasonication (99 times 3 s cycle, 100 W, 0 °C). The supernatant containing crude proteins was collected after centrifugation at 15 000 × *g* for 1 h at 4 °C. The MLs were washed with sterile saline and cultured in RPMI-1640 medium (5000 worms/mL) at 37 °C and 5% CO_2_ for 18 h. The culture medium containing ML ES proteins was filtered with a 0.22 μm membrane and concentrated using an ultrafiltration tube. The concentrations of the crude and ES proteins were assessed by the Bradford method.

### Bioinformatics analysis of TsSerp

The full-length cDNA sequences of the TsSerp gene were retrieved from GenBank (GenBank: AY028974.1). The physicochemical characteristics of TsSerp were analysed by using the online website for ExPASy (https://www.expasy.org/), using bioanalysis software and websites. The signal peptide and subcellular localization were predicted as previously reported [31]. The tertiary structure of TsSerp was predicted by PyMOL software, and its functional sites were analysed using CN3D software [32]. The amino acid sequence of the TsSerp gene was compared with that of serine proteases from other organisms with Clustal X. The GenBank accession numbers of serine proteases from other organisms were as follows: *Trichinella nativa* (KRZ59611.1), *Trichinella britovi* (KRY58838.1), *Trichinella pseudospiralis* (ABY73337.1), *Trichinellla murrelli* (KRX47710.1), *Trichinella* sp. T6 (KRX77113.1), *Trichinella nelsoni* (KRX25903.1), *Trichinella* sp. T8 (KRZ96358.1), *Trichinella* sp. T9 (RX62553.1), *Trichinella papuae* (KRZ79030.1), *Trichinella zimbabwensis* (KRZ18689.1), *Trichinella patagoniensis* (KRY23880.1), *Mus musculus* (AAA40105. 1) and *Homo sapiens* (AAF21966.1). The phylogenetic analysis was conducted with MEGA 7.0 based on the neighbour-joining (NJ) method, as previously described [33].

### Cloning, expression and identification of TsSerp

Total RNA was isolated from MLs using TRIzol (Invitrogen, USA). The full-length TsSerp cDNA sequence was amplified by PCR using specific primers carrying restriction enzyme sites for *Bam*HI and *Hind*III **(bold**) (5′-GC**GGATCC**CAGTATTGTGGAAATCCTTATTTT-3′; GCGGCG**AAGCTT**TCAGTAAAAAGAGTCAAAATT’). The PCR products were cloned into the expression vector pET-32a, and then the recombinant pET-32a/TsSerp plasmid was transformed into *Escherichia coli* BL21 (DE3) (Novagen, USA). rTsSerp was expressed by induction with 0.5 mM IPTG for 4 h at 25 °C, with the formation of insoluble inclusion bodies [34]. The inclusion bodies were recovered from the bacterial lysates by centrifugation at 12,000 × *g* for 10 min and dissolved in 8 M urea. rTsSerp was purified using Ni–NTA-Sefinose resin kit (Sangon Biotech Co., Shanghai, China). After purification, the purified rTsSerp was renatured by gradient dialysis [35, 36]. The protein concentration of purified rTsSerp was determined and analysed by SDS-PAGE and western blotting as previously reported [37].

### Immunization of mice and ELISA determination of anti-rTsSerp antibodies

Two hundred mice were randomly divided into four groups (50 animals per group). Each mouse was subcutaneously immunized with 20 µg of rTsSerp emulsified with ISA 201 adjuvant and boosted three times with rTsSerp with ISA 201 (Seppic, France) at a 2-week interval. Control groups received TRX-tag + ISA 201, only ISA 201 adjuvant or PBS alone at the same time intervals as the experimental groups [38]. One hundred microlitres of blood was collected from the tail of each mouse at weeks 0, 2, 4, 6 and 8 after immunization, and individual serum samples were isolated and stored at − 80 °C until use [39].

Specific anti-rTsSerp antibody responses (total IgG, IgG1 and IgG2a) in all vaccinated mice were measured by indirect ELISA with rTsSerp [40]. The IgE and IgM responses were also determined by ELISA. Briefly, the ELISA plate was coated with 2.5 μg/mL rTsSerp at 4 °C overnight. After washing with PBS (pH 7.4) containing 0.05% Tween-20 (PBST), the plate was blocked with 5% nonfat milk at 37 °C for 2 h. After washing with PBST, the plates were incubated at 37 °C for 2 h with a 1:100 dilution of murine immune serum, followed by incubation with HRP-conjugated anti-mouse IgG (IgG1 and IgG2a; 1:5000 dilutions), IgE and IgM (1:2500 dilutions; Sigma) for 1 h at 37 °C. Plates were developed with OPD (Sigma) plus H_2_O_2_, and the reaction was finished by the addition of 2 M H_2_SO_4._ The OD values at 492 nm were measured by a microplate reader (Tecan, Schweiz, Switzerland) [28, 41].

### Identification of rTsSerp antigenicity by western blotting

ML crude proteins, ML ES proteins and rTsSerp were separated by 12% SDS-PAGE. The proteins were transferred onto a nitrocellulose membrane (Millipore, USA) [33, 42]. The membrane was blocked with 5% nonfat milk diluted in Tris-buffered saline-0.5% Tween-20 (TBST) at 37 °C for 2 h and cut into strips. The strips were probed with 1:100 dilutions of various sera (anti-rTsSerp serum, infected mouse serum and normal mouse serum) at 4 °C overnight. After washing with TBST, the strips were incubated with HRP-conjugated anti-mouse IgG (1:5000; Southern Biotechnology, USA) at 37 °C for 1 h. After washing again, the strips were developed with 3,3′-diaminobenzidine tetrahydrochloride (DAB; Sigma-Aldrich), and the reaction was terminated by washing the membrane with deionized water [31].

To assess the relative TsSerp protein expression in diverse *T. spiralis* stages, 8 μg/lane crude ML, IIL, 3 dpi AW and NBL proteins were analysed by SDS-PAGE and western blotting with 1:100 dilutions of anti-rTsSerp serum [43]. A mouse antibody against GAPDH (1:1000) was used to ascertain GAPDH protein expression as an internal control [44]. After washing with TBST, the strips were coloured with an enhanced chemiluminescent kit (CWBIO, Beijing, China) [23]. The relative TsSerp protein expression in various *T. spiralis* stages was analysed by ImageJ software.

### RT-PCR analysis of TsSerp mRNA expression

Total RNA from NBLs, MLs, IILs and 3-day AWs was extracted using TRIzol reagent (Invitrogen, USA). RT-PCR was performed to investigate the transcription levels of the TsSerp gene in various *T. spiralis* phases as described before [19]. The *T. spiralis* housekeeping gene GAPDH (GenBank: AF452239) was also amplified and used as an internal control [45]. PBS was utilized as a negative control in all PCR amplifications.

### Immunofluorescence assay (IFA)

Fresh whole worms of diverse *T. spiralis* phases (ML, IIL, AW and NBL) were fixed with 4% neutral formaldehyde for 30 min and then re-fixed with cold acetone for 20 min. Moreover, the worms were embedded in paraffin, and 3-µm-thick cross-sections were cut with a microtome. The expression and worm tissue location of native TsSerp in diverse *T. spiralis* stages were observed using IFA as previously reported [46, 47]. Briefly, intact whole nematodes and cross-sections were blocked with 5% normal goat serum in PBS and then probed at 37 °C for 2 h with 1:10 dilutions of various sera (anti-rTsSerp serum, infection serum or pre-immune serum). After washing with PBS, the samples were incubated with FITC-conjugated goat anti-mouse IgG (1:100; Santa Cruz, USA). After washing again, the whole worms and cross-sections were observed under a fluorescence microscope (Olympus, Japan) [48].

### Assay of TsSerp-specific cytokines

To assess the TsSerp-specific cellular immune responses, ten vaccinated mice from each group were sacrificed at weeks 0 and 8 following immunization and 2 weeks after challenge infection. Murine spleens and mesenteric lymph nodes (MLNs) were collected and homogenized in complete RPMI-1640 medium (Gibco, Auckland, New Zealand). Pellets were obtained after centrifugation at 300 × *g* for 5 min, and the cells were isolated as previously described [21, 49]. The cell density was set to 2 × 10^6^ cells/mL in RPMI-1640 medium with 5% foetal bovine serum (FBS), penicillin (100 U/mL) and streptomycin (100 μg/mL). These cells were stimulated using 10 μg/mL rTsSerp for 72 h at 37 °C and 5% CO_2_. The supernatant was collected, and three cytokines (IFN-γ, IL-4 and IL-2) were measured using a conventional sandwich ELISA [39, 50]. The concentrations of cytokines are presented as picograms per millilitre (pg/mL).

### In vitro larval invasion into IECs

To ascertain the role of TsSerp in larval invasion into the enteric epithelium, an in vitro invasion test was performed as previously reported [8, 51]. Briefly, the MLs were first activated into IILs by using 5% swine bile at 37 °C for 2 h, and 100 IILs suspended in semisolid medium were placed onto the IEC monolayer [52]. The medium was pre-replenished with serially diluted rTsSerp protein (0, 2.5, 5.0, 7.5, 12.5 and 15.0 μg/mL) or serial dilutions (1:100–1:1000) of anti-rTsSerp serum, infection serum or pre-immune serum. Following culture at 37 °C for 2 h, larval invasion of the IECs was observed under a microscope. The IILs that invaded the IEC monolayer and migrated were designated invaded larvae, whereas the larvae that still stayed on the surface of the IEC monolayer and remained in the coil form were designated non-invaded larvae [53]. Each test was performed in triplicate.

### rTsSerp facilitation or anti-rTsSerp serum suppression of larval invasion on excised intestine

The rTsSerp function in larval invasion of enteric epithelia was also assessed using the excised murine small intestine as previously reported [54]. One hundred IILs were first mixed with a 1:100 dilution of anti-rTsSerp serum or anti-TRX-tag serum, 20 μg/mL rTsSerp or TRX-tag, or PBS. Small intestines were excised from normal BALB/c mice, washed with sterilized Tyrode’s solution, and then cut into 2-cm-long segments. Two ends of the segment were ligated to form an intestinal pouch, and the IILs were injected into the bowel lumen and maintained in sterilized Tyrode’s solution for 2 h at 37 °C. Each test was performed in triplicate, and the larvae in the gut lumen were designated non-invaded worms [54].

### Antibody-dependent cellular cytotoxicity (ADCC) test

Anti-rTsSerp antibody cytotoxicity on the NBLs was assessed as previously described [55, 56]. Forty NBLs were cultured at 37 °C for 12–48 h with 2 × 10^5^ mouse peritoneal exudate cells (PECs) in 96-well plates in RPMI-1640 medium supplemented with 1:10–1:200 dilutions of anti-rTsSerp serum, infection serum or pre-immune serum. Each test was performed in triplicate. The larval viability following ADCC was assessed based on larval morphology and activity. The living NBLs were active and showed mobility, whereas the dead larvae were straight and inactive [34]. The cytotoxicity was ascertained as the percent of the dead larvae to the total larvae observed in each test [22].

### Challenge infection and evaluation of immune protection

To assess the immune protection induced by vaccination with rTsSerp, each mouse among all the vaccinated mice was inoculated orally with 300 *T. spiralis* MLs two weeks after the last vaccination. The AWs were collected from the small intestines of ten vaccinated mice from each group at 6 dpi [57]. The MLs were harvested by artificial digestion of the skeletal muscles of an additional ten vaccinated mice from each group at 56 dpi. The immune protective efficacy elicited by TsSerp vaccination was ascertained as the reduction of intestinal AWs and muscle larvae per gram (LPG) of tissue from vaccinated mice compared to those of the PBS group mice [50].

### Muscle histopathological examination

At 56 dpi, the masseter muscles were excised from infected mice, fixed in 4% formalin for 24 h and embedded in paraffin wax. Three-micrometre-thick muscle sections were prepared, deparaffinized and stained using haematoxylin and eosin (HE). The sections were examined under microscopy, and the inflammatory cells (eosinophils and lymphocytes) per field (200 ×) were counted.

### Statistical analysis

All the data were statistically analysed with SPSS for Windows, version 20.0. The data are shown as the mean ± standard deviation (SD). After being tested by Shapiro–Wilk's test and Levene's test to check the datum normality and homogeneity, differences among different groups were analysed by a Chi-square test or one-way ANOVA. Correlation analysis was used to assess the relationship between ADCC and serum dilution/culture time. *P* < 0.05 was considered statistically significant.

## Results

### Bioinformatics analysis of TsSerp

**Figure 1:**
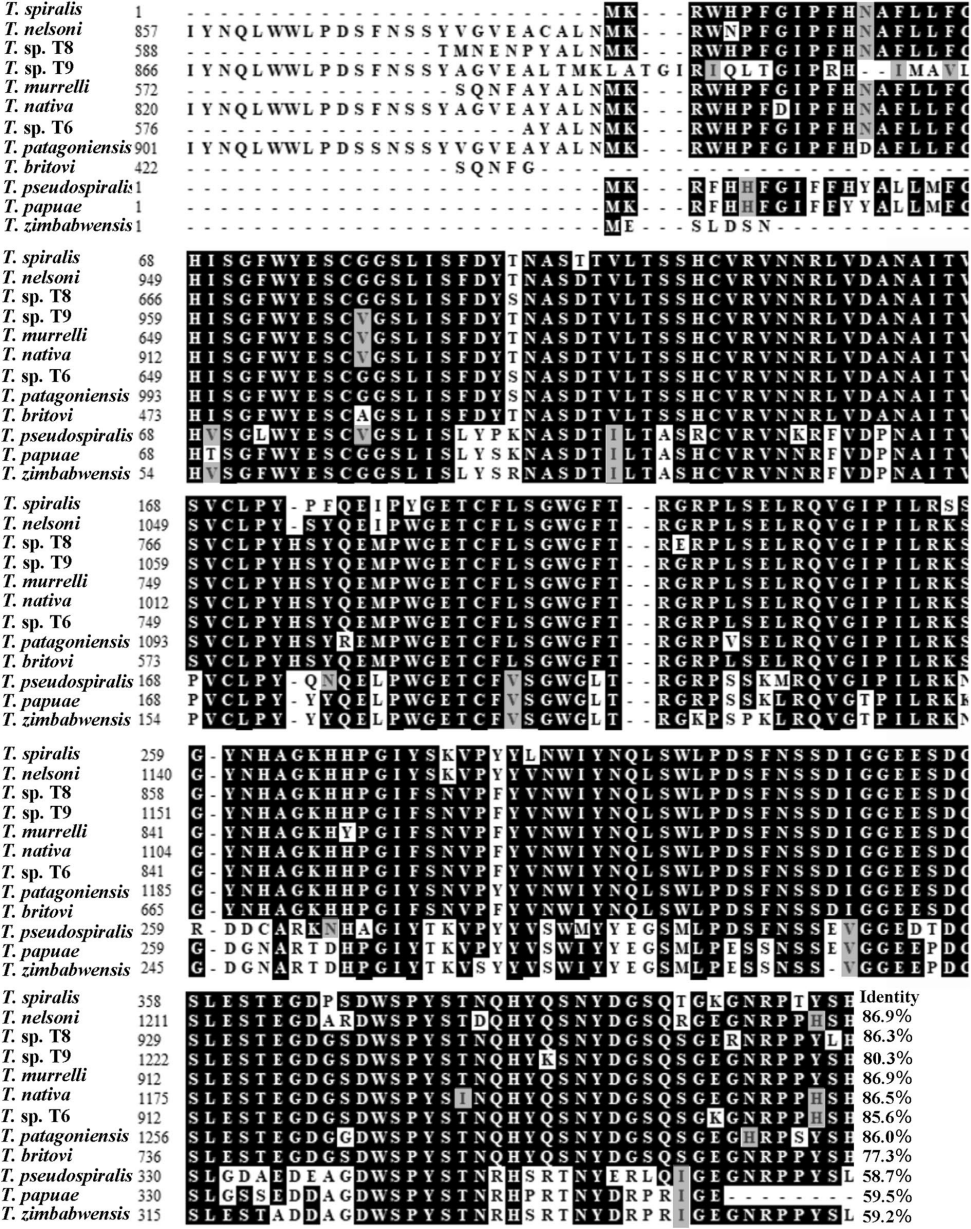
**Sequence alignment of the** ***Trichinella spiralis*** **serine protease gene (AY028974.1) with those of other species or genotypes of the genus** ***Trichinella***. Clustal X and BOXSHADE were used to analyse the sequences, and distinct differences were observed in various *Trichinella* species/genotypes. Black shading indicates residues identical to TsSerp, and grey shading shows conservative substitutions.

The full-length cDNA sequence of the TsSerp gene is 1445 bp, encoding 421 aa with a molecular weight (MW) of 48 kDa and isoelectric point (pI) of 6.33. TsSerp has a signal peptide at residues 1–27. Subcellular localization suggested that TsSerp is a secretory protein. The homology comparison of TsSerp amino acid sequences with those of serine proteases of other *Trichinella* species or genotypes is shown in Figure 1. The amino acid sequence of TsSerp had an identity of 86.9, 86.3, 80.3, 86.9, 85.6, 86.5, 86.0 and 77.3% with those of the serine proteases of the 8 encapsulated *Trichinella* species (*T. nelsoni,* T8, T9, *T. murrelli, T. nativa*, T6, *T. patagoniensis* and *T. britovi*), and it had an identity of 58.7, 59.2 and 59.5% of those from 3 non-encapsulated *Trichinella* species (*T. pseudospiralis*, *T. zimbabwensis* and *T. papuae*).

**Figure 2:**
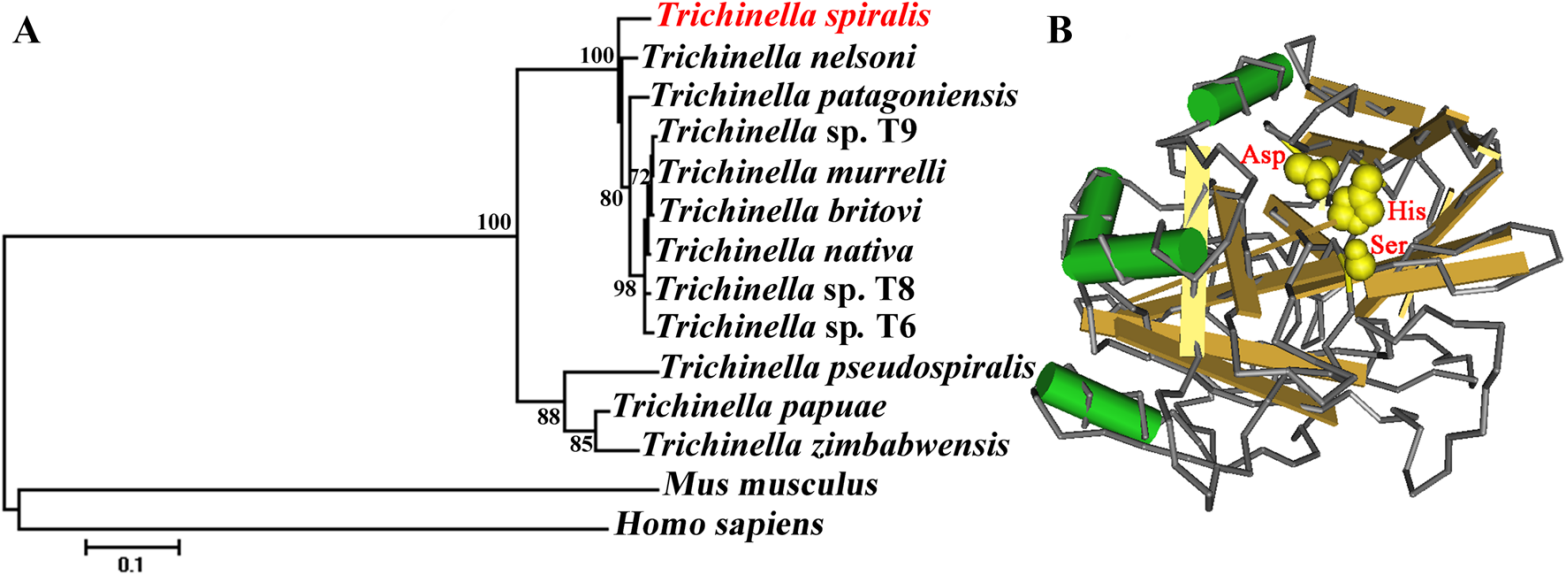
Phylogenetic trees of serine proteases of 14 organisms produced with the NJ method (**A**) and the predicted 3-dimensional structure of the TsSerp protein (**B**). Three serine protease-specific active site residues (His, Asp and Ser) of TsSerp are marked yellow.

Phylogenetic analysis of TsSerp with serine proteases of other *Trichinella* species or genotypes is shown in Figure 2A. The phylogenetic tree showed that a monophyletic group of the genus *Trichinella* was well supported. Within the genus *Trichinella*, two clear clades were present: one was the encapsulated clade (including *T. spiralis, T. nelsoni, T. patagoniensis,* T9, *T. murrelli, T. britovi, T. nativa,* T8 and T6), and the other was the non-encapsulated clade (*T. pseudospiralis*, *T. papuae* and *T. zimbabwensis*).

Structure prediction showed that TsSerp has 15 α-helices, 9 β-strands, and a domain (between positions 47 and 281 aa) of a trypsin-like serine protease with an active site containing the classic catalytic triad. In the three-dimensional model, the catalytic triad serine–histidine–aspartate motif formed a functional domain with substrate-binding sites (Figure 2B).

### Expression and identification of rTsSerp protein

**Figure 3:**
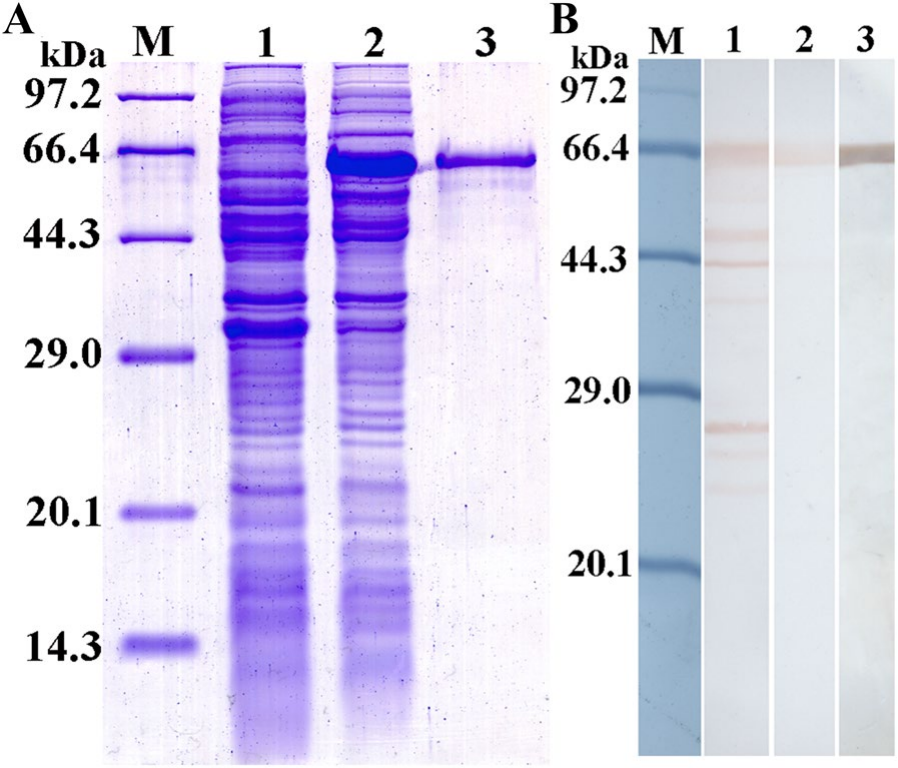
**Identification of rTsSerp. A** SDS-PAGE analysis of rTsSerp. Lane M: protein marker; lane 1: lysate of recombinant *E. coli* carrying pET-32a/TsSerp prior to induction; lane 2: lysate of recombinant *E. coli* carrying pET-32a/TsSerp after induction; lane 3: purified rTsSerp. **B** Identification of rTsSerp by western blotting. Lane M: protein marker; lane 1: lysate of recombinant *E. coli* carrying pET-32a/TsSerp after induction was recognized by serum from infected mice. Purified rTsSerp (lanes 2 and 3) was identified by infection serum (lane 2) and an anti-his-tag monoclonal antibody (lane 3).

The SDS-PAGE results showed that the BL21 bacteria carrying pET-32a/TsSerp expressed a fusion protein with a molecular weight (MW) of 65.7 kDa. The MW of the rTsSerP protein was consistent with the predicted combined size of the TsSerp protein encoded by the cDNA clone (44.7 kDa) and the TRX + his-tag (21 kDa) from the vector (Figure 3A). Western blotting analysis indicated that rTsSerp was recognized by the serum of *T. spiralis*-infected mice and an anti-his-tag monoclonal antibody (Figure 3B).

### Identification of rTsSerp antigenicity

**Figure 4:**
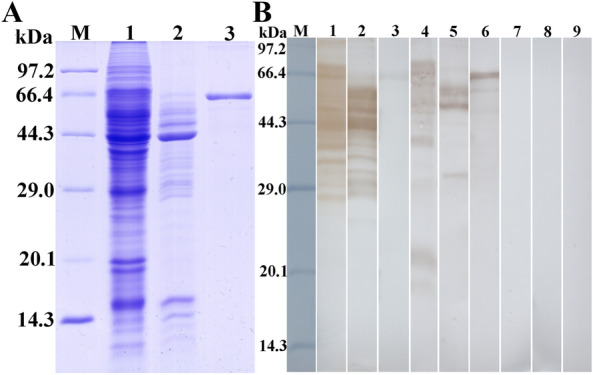
**Identification of rTsSerp antigenicity. A** SDS-PAGE analysis of rTsSerp. Lane M: protein marker; lane 1: ML crude proteins; lane 2: ML ES proteins; lane 3: rTsSerp. **B** Western blotting of rTsSerp antigenicity. ML crude proteins (lane 1), ML ES proteins (lane 2) and rTsSerp (lane 3) were recognized by infection serum; the native TsSerp in ML crude proteins (lane 4) and ML ES proteins (lane 5) and rTsSerp (lane 6) were identified by anti-rTsSerp serum; and ML crude (lane 7) and ES proteins (lane 8), and rTsSerp (lane 9) were not detected by normal mouse pre-immune serum.

SDS-PAGE showed that after purification, the rTsSerp protein exhibited a clear single protein band (Figure 4A). Western blotting results revealed that rTsSerp was recognized by anti-rTsSerp serum and infection serum but not by normal mouse serum (Figure 4B). The native TsSerp at 18.8–83.7 kDa in ML crude proteins as well as ML ES proteins was also recognized by anti-rTsSerp serum and infection serum but not by normal mouse serum, suggesting that native TsSerp is a secretory protein.

### TsSerp mRNA and protein expression in diverse *T. spiralis* phases

**Figure 5:**
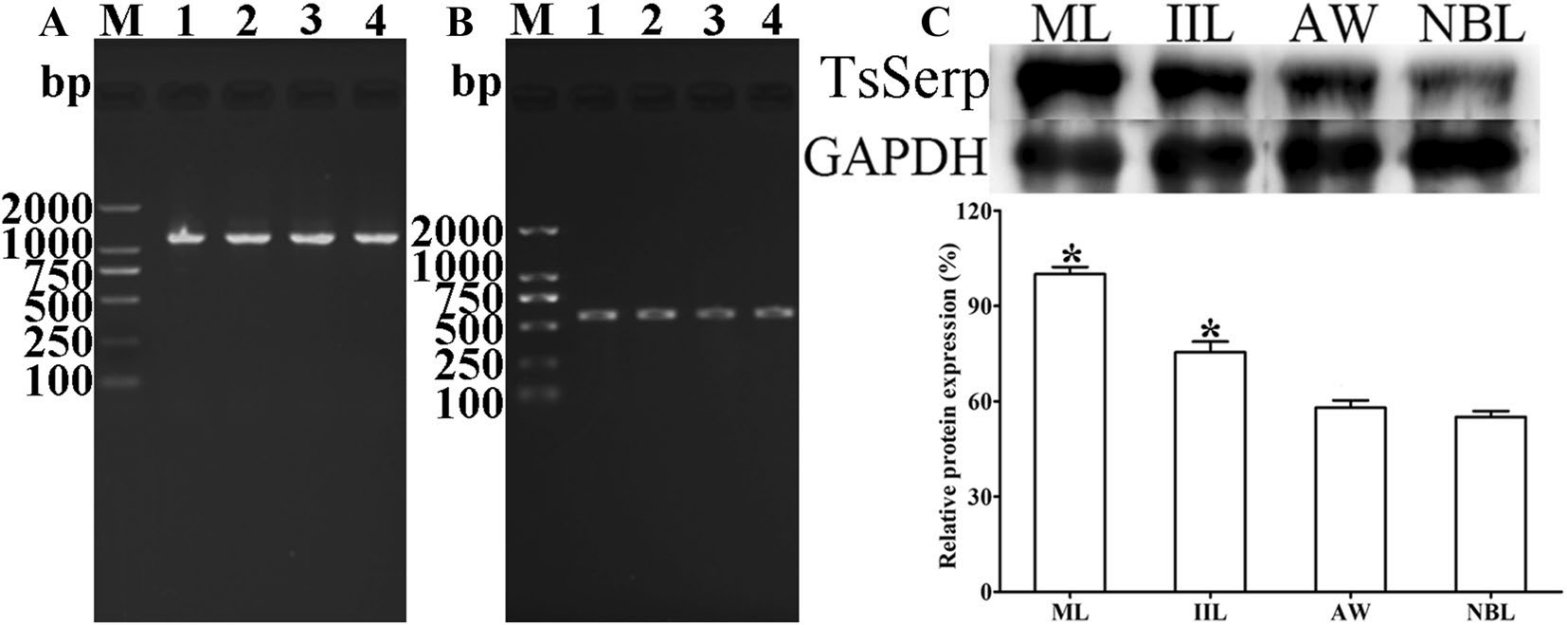
**TsSerp mRNA and protein expression in diverse** ***T. spiralis*** **phases. A,B** RT-PCR analysis of transcription of TsSerp (**A**) and GAPDH (**B**) at various phases. Lane M: DNA marker; lane 1: ML; lane 2: IIL; lane 3: 3-day AW; lane 4: NBL. **C** Western blotting analysis of TsSerp protein expression levels at diverse stages. The histogram shows the relative TsSerp protein expression levels assessed by densitometry from three independent tests. *P < 0.001 compared with the AW and NBL groups.

RT-PCR results showed that TsSerp transcription was observed at all four developmental phases of *T. spiralis* (Figure 5A), and the housekeeping gene (GAPDH) also generated the expected size (570 bp) in all four lifecycle phases (Figure 5B). Western blotting results revealed that anti-rTsSerp serum identified the native TsSerp protein in crude proteins of various *T. spiralis* phases (ML, IIL, 3 dpi AW and NBL) (Figure 5C), demonstrating that TsSerp was expressed at diverse *T. spiralis* lifecycle phases. The relative quantitative results showed that the TsSerp protein expression level in MLs and IILs was obviously higher than that in the other two stages (AW and NBL; *χ*^2^ = 62.405, *P* < 0.001).

### Expression and worm tissue localization of native TsSerp protein in diverse *T. spiralis* stages

**Figure 6:**
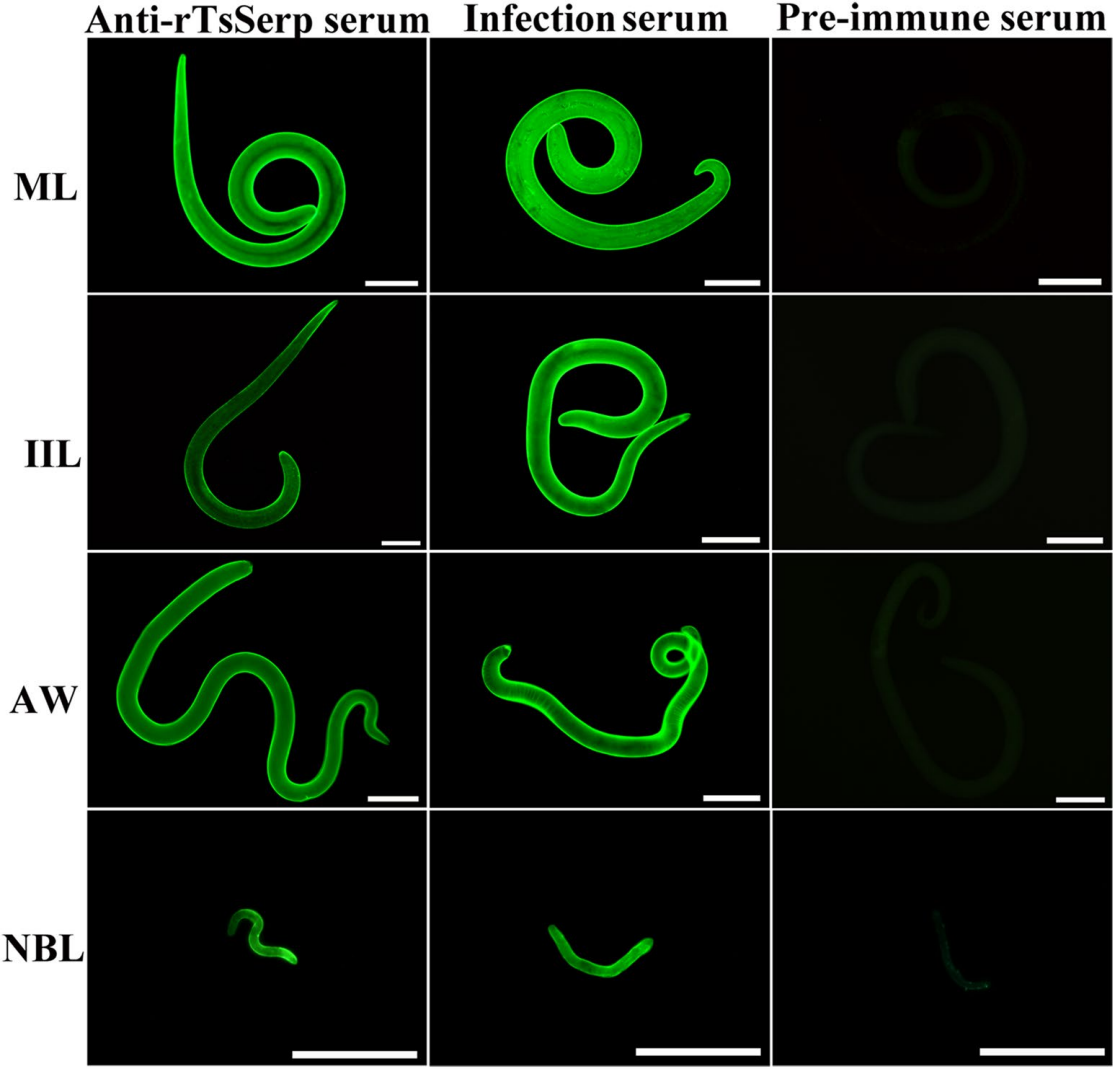
**Expression of TsSerp at the cuticle of various** ***T. spiralis*** **stages by IFA**. Whole worms were probed by anti-rTsSerp serum, and immunostaining was observed at the epicuticle of MLs, IILs, AWs and NBLs. However, pre-immune serum did not recognize any worm components of the parasite. Scale bars of MLs, IILs and AWs = 100 μm; Scale bars of NBLs = 50 μm.

**Figure 7:**
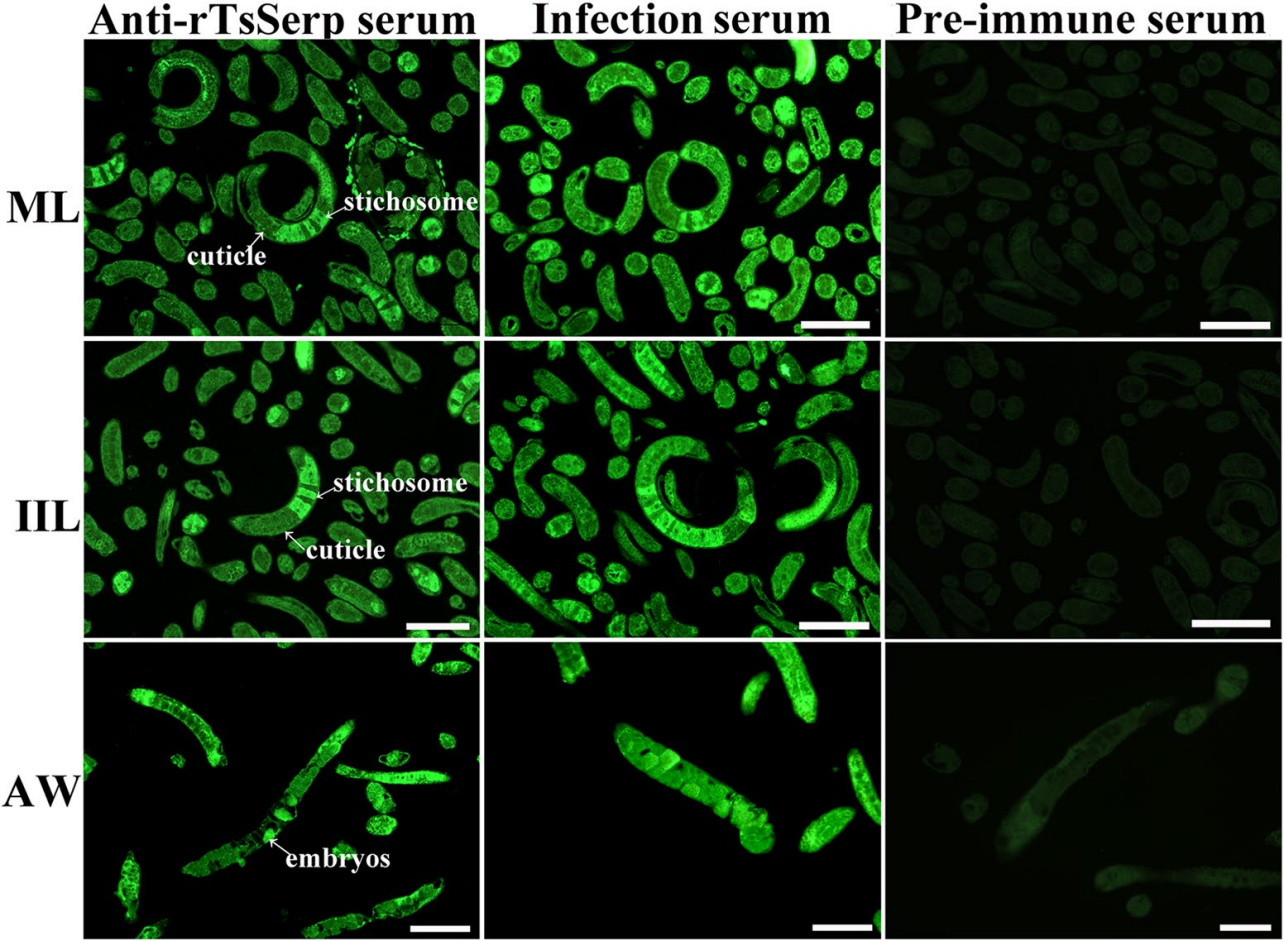
**Immunolocalization of TsSerp in cross-sections of diverse** ***T. spiralis*** **stages by IFA with anti-rTsSerp serum**. Fluorescence staining was observed at the cuticle and stichosome of MLs, IILs and intrauterine embryos of the adult females. No immunostaining in cross-sections was observed by using pre-immune serum as a negative control. Scale bars: 100 μm.

The results of IFA with whole parasites revealed green immunostaining on the epicuticle of MLs, IILs, AWs and NBLs by using anti-rTsSerp serum and infection serum (Figure 6). When the worm cross-sections were probed by anti-rTsSerp serum, the immunostaining was located at the cuticle and stichosome of MLs and IILs and intrauterine embryos of the female adults (Figure 7). No worm tissue components of the nematode were identified by pre-immune serum.

### Anti-TsSerp antibody response to rTsSerp immunization

**Figure 8:**
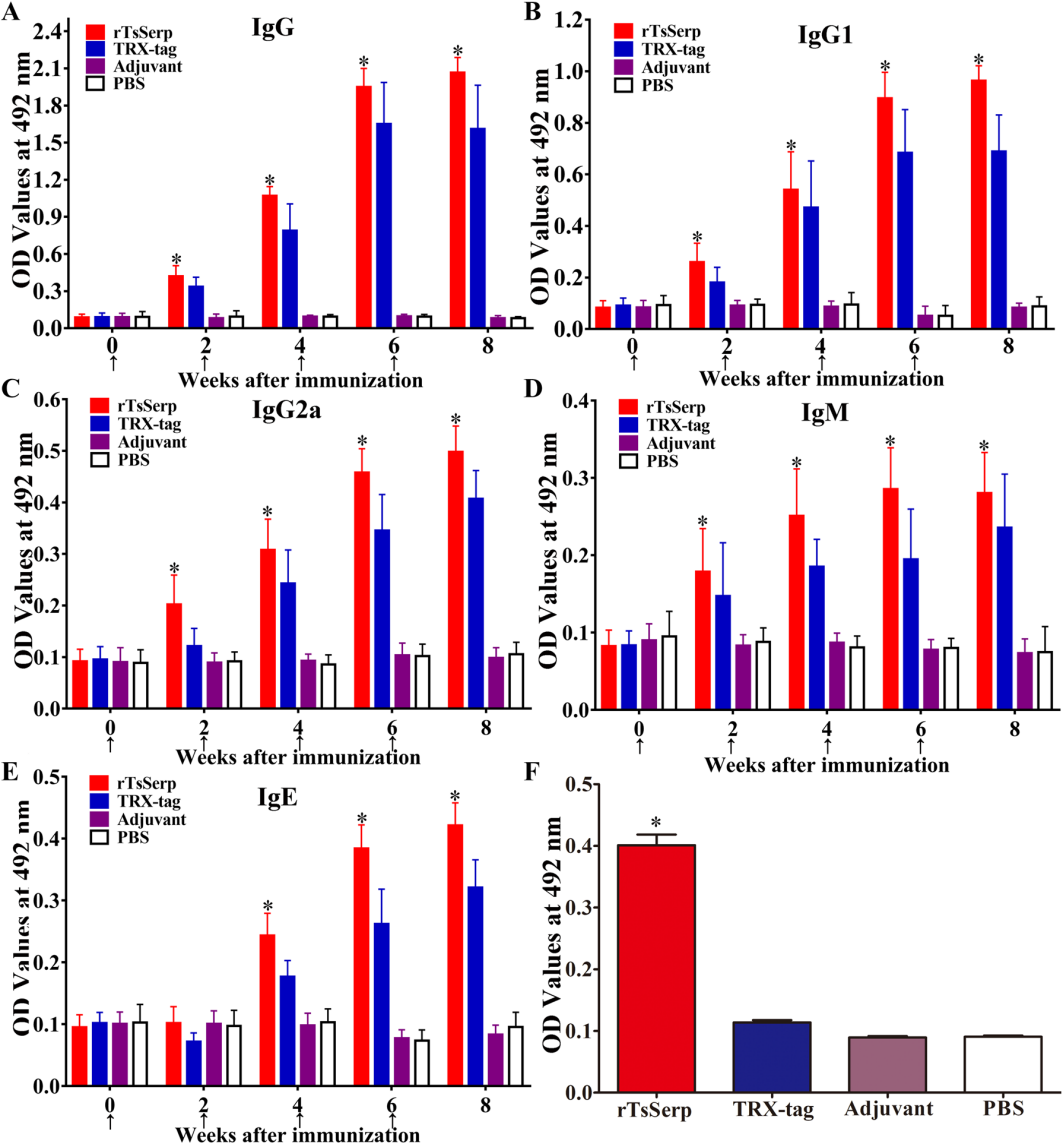
**Specific antibody responses in mice vaccinated with rTsSerp. A** Specific total IgG in mice vaccinated with rTsSerp or control mice (TRX-tag, ISA 201 adjuvant and PBS) at various time intervals after vaccination. Specific IgG1 (**B**) and IgG2a (**C**) subclass responses against rTsSerp at various times after vaccination. Specific IgM (**D**) and IgE (**E**) levels in vaccinated mice. **F** Anti-rTsSerp IgG in mice vaccinated with rTsSerp or TRX-tag at two weeks after the last immunization was also measured by ELISA with ML ES antigens. The OD values from each group are shown as the mean ± SD of antibody levels (n = 20). The vaccination times are indicated with arrows (↑). *P < 0.001 compared with the adjuvant or PBS group.

To assay specific anti-rTsSerp antibody responses, rTsSerp-specific IgG, IgG1, IgG2a, IgM and IgE in serum samples from vaccinated mice were determined by rTsSerp-ELISA. The mice were vaccinated using rTsSerp four times, the anti-rTsSerp IgG level in vaccinated mice was evidently elevated after the second vaccination, and anti-rTsSerp IgG titres at two weeks after the last immunization reached 1:10^5^, indicating that rTsSerp is immunogenic. However, no mice inoculated with only ISA 201 adjuvant or PBS alone exhibited any anti-rTsSerp IgG responses (Figure 8A). The IgG1 level on weeks 4, 6 and 8 after immunization was significantly higher than the IgG2a level (*t*_4w_ = 6.529, *t*_6w_ = 12.391, *t*_8w_ = 27.222, *P* < 0.0001) (Figure 8B, C), demonstrating that immunization with rTsSerp triggered a Th2-predominant mixed Th1/Th2 response. Furthermore, anti-rTsSerp IgM and IgE were also ascertained, and the results revealed that specific IgM and IgE levels were obviously elevated in mice immunized with rTsSerp compared with those in ISA 201 and PBS control group mice (*F*_IgM_ = 166.592, *F*_IgE_ = 1031.051, *P* < 0.0001) (Figure 8D, E), suggesting that specific anti-rTsSerp IgE is likely to play a vital role in TsSerp-produced rapid expulsion of adult worms from the gut.

Additionally, serum anti-rTsSerp IgG in mice vaccinated with rTsSerp or TRX-tag at two weeks after the final immunization was also measured by ELISA with ML ES antigens based on rTsSerp carrying with the TRX-tag. The results showed that ML ES antigens were not recognized by sera from mice vaccinated with TRX-tag alone (Figure 8F), and the OD values between the rTsSerp and TRX-tag groups were significantly different (*r* = 0.935, *P* < 0.0001).

### Cytokine responses to vaccination with rTsSerp

**Figure 9:**
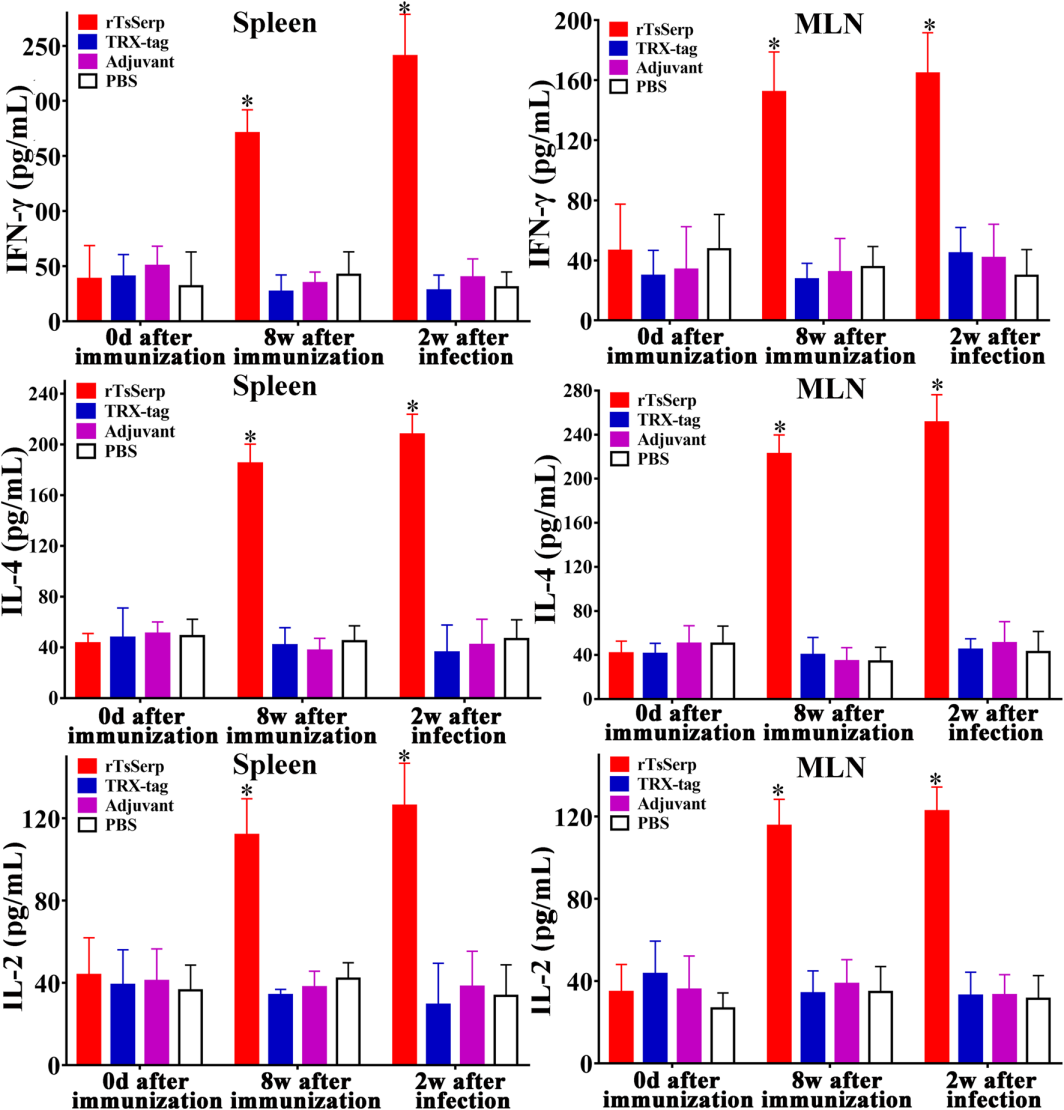
**Cytokines secreted by the spleen and mesenteric lymph nodes (MLNs) from mice vaccinated with rTsSerp at various times after vaccination.** The concentrations of three cytokines (IFN-γ, IL-4 and IL-2) were measured in the supernatant after the spleen and MLN cells were stimulated with 10 μg of rTsSerp for 72 h. The data are presented as the mean ± SD of ten mice per group. *P < 0.0001 in comparison with the TRX-tag, ISA 201 adjuvant and PBS control groups

To ascertain the cytokine responses elicited by rTsSerp vaccination, spleen and MLN cells collected from vaccinated mice were cultured under rTsSerp stimulation. The supernatant was obtained, and the cytokine concentration was assayed by sandwich ELISA. The levels of Th1 cytokines (IFN-γ) and Th2 cytokines (IL-4) were significantly elevated at 8 weeks after immunization with rTsSerp and 2 weeks after challenge compared to those in the TRX-tag, ISA 201 adjuvant and PBS groups (*P* < 0.0001) (Figure 9). Moreover, the IL-2 level of immunized mice at 8 weeks after immunization and 2 weeks after challenge was also evidently higher than that in the mice in the three control groups (*P* < 0.0001). Our results demonstrated that rTsSerp vaccination triggered mixed Th1/Th2 responses on the basis of specific IgG subclass responses and cytokine generation, suggesting that subcutaneous vaccination with rTsSerp triggered both systemic (spleen) and local intestinal mucosal (MLN) cellular immune responses.

### rTsSerp facilitation or anti-rTsSerp serum suppression of larval invasion of IECs

**Figure 10:**
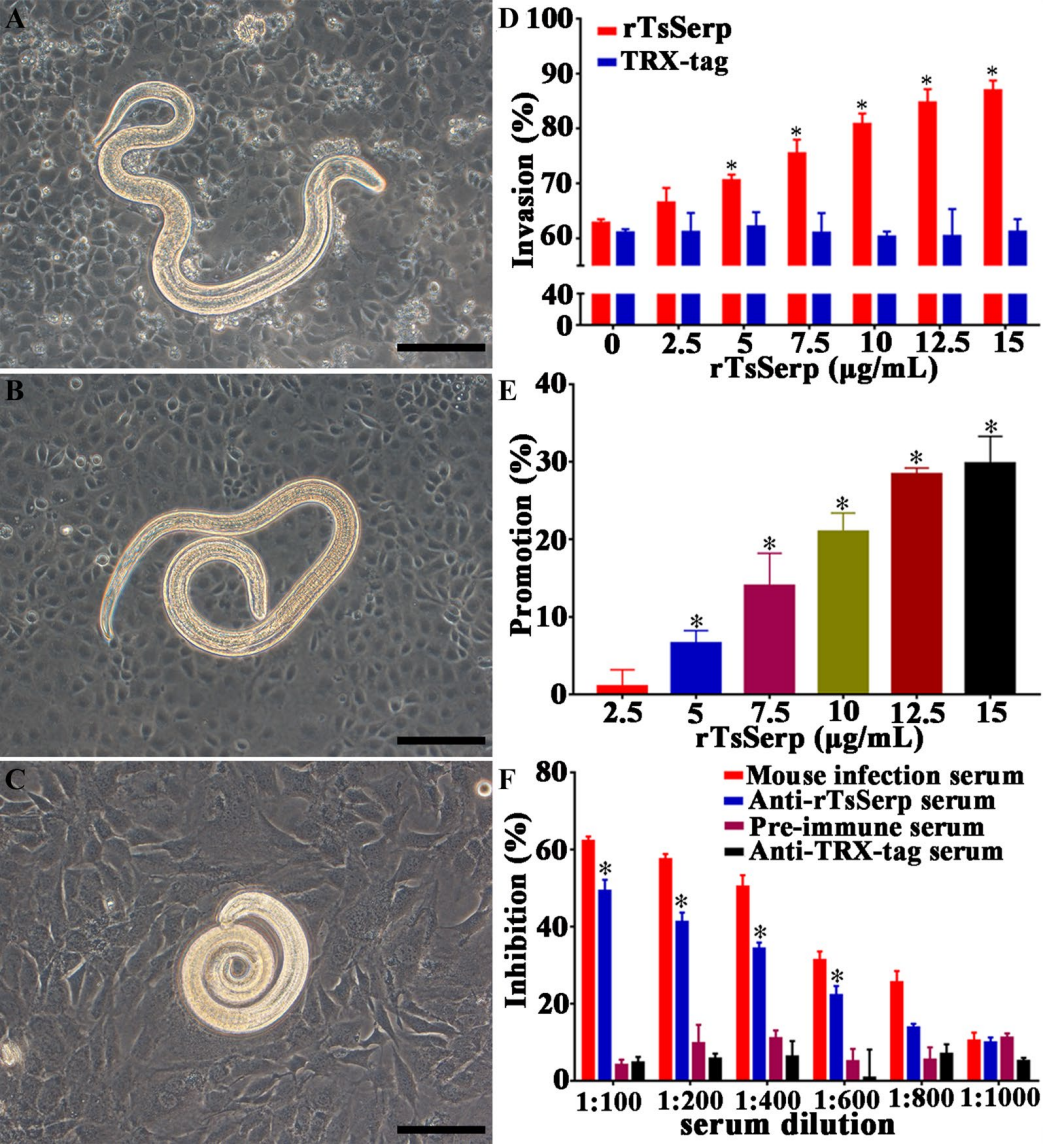
**rTsSerp facilitation or anti-rTsSerp serum suppression of larval invasion of IECs.** *Trichinella spiralis* MLs were first activated into IILs using 5% swine bile for 2 h at 37 °C, the IILs were added onto the IEC monolayer, and the IILs that invaded the IECs were observed under microscopy after culture for 2 h (400 ×). **A** Larvae invading the IEC monolayer were mobile and migratory. **B** Non-invaded IILs coiled on the surface of the IEC monolayer. **C** Non-invaded IILs coiled on the surface of C2C12 cells, which were not sensitive to larval invasion and utilized as negative control cells**. D** and **E** rTsSerp facilitation of IIL invasion of IECs. **F** Anti-rTsSerp serum suppression of IIL invasion of IECs. *P < 0.01 compared to TRX-tag, anti-TRX-tag serum and pre-immune serum. Scale bars: 100 μm.

After culture with an IEC monolayer for 2 h, the IILs that invaded into the monolayer were observed (Figure 10A-C). When the medium was replenished with rTsSerp-supplemented medium and the IECs were cultured with the IILs for 2 h, rTsSerp obviously facilitated the larval invasion of the IECs; this facilitation was rTsSerp dose dependent (*r* = 0.996) and showed a positive trend with the increase in the rTsSerp concentration (*F* = 425.376, *P* < 0.001); however, the TRX-tag did not accelerate larval invasion (Figure 10D, E). When serial dilutions of anti-rTsSerp serum were supplemented into the culture medium and incubated with IECs for 2 h, anti-rTsSerp serum (1:100–1:600) resulted in significant suppression of larval invasion compared to that in the PBS group (*P* < 0.01). The suppression was dose dependent for anti-rTsSerp antibodies (*r* = 0.986) and showed a decreasing trend with increasing serum dilutions (*F* = 181.170, *P* < 0.0001) (Figure 10F). Moreover, anti-TRX-tag serum and pre-immune serum did not show any suppression effects on the larval invasion of the IECs.

### rTsSerp facilitation or anti-rTsSerp serum suppression of larval invasion of excised intestine

**Figure 11:**
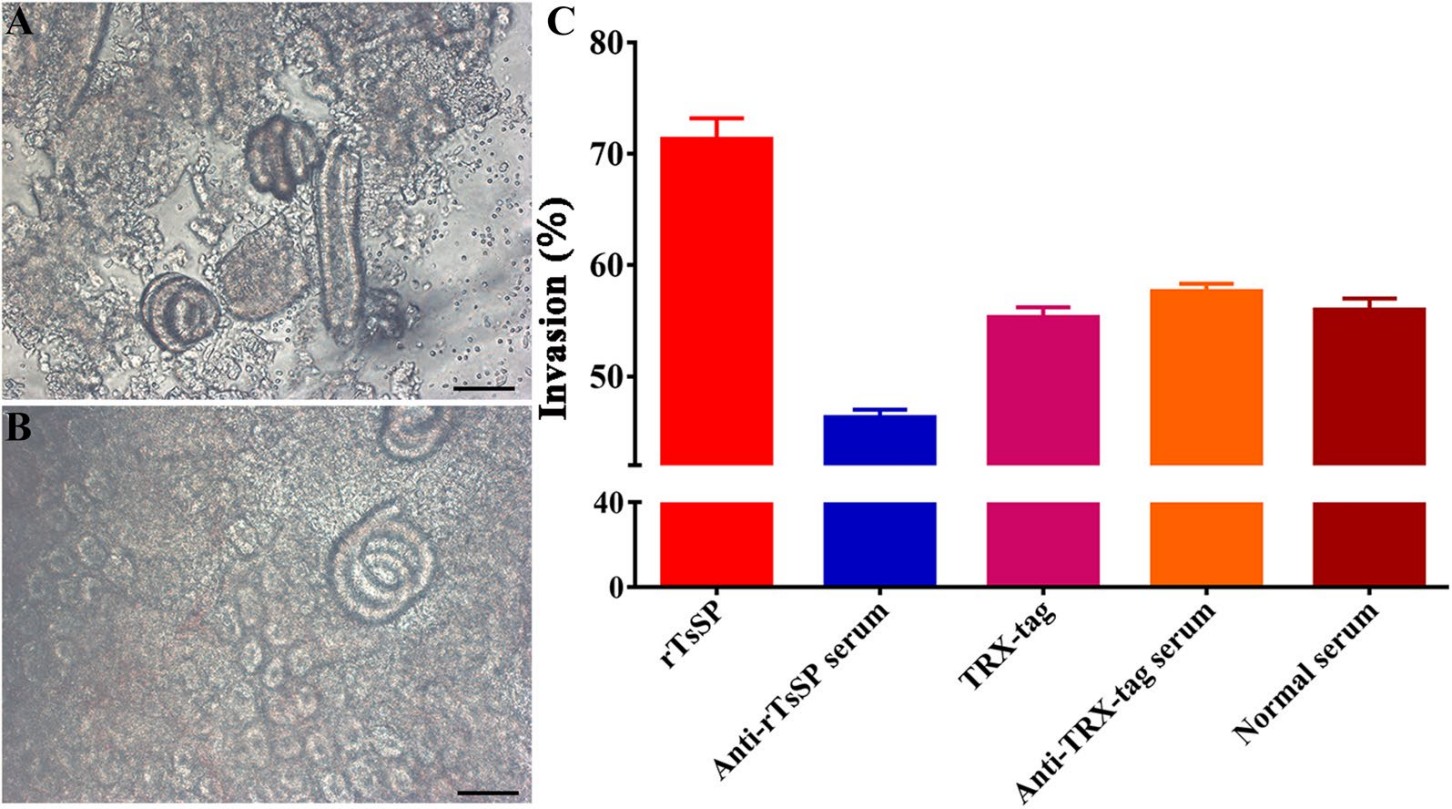
**rTsSerp facilitation or anti-rTsSerp serum suppression of larval invasion of excised intestine.** One hundred IILs were pre-incubated with rTsSerp, TRX-tag, anti-rTsSerp serum, anti-TRX-tag serum or PBS, subsequently injected into excised murine intestinal lumen, and maintained in Tyrode’s solution at 37 °C for 2 h. The enteric segment was cut off, and the intestinal mucosa was compressed between two thick slides and examined under microscopy. **A** Non-invaded larvae were spiral coils within the enteric lumen (200 ×). **B** Invaded larvae were inside the intestinal mucosa (400 ×). **C** The invasion rate of excised intestine by larvae pre-incubated with rTsSerp or anti-rTsSerp serum. *P < 0.0001 compared to the TRX-tag, anti-TRX-tag serum or PBS group. Scale bars: 100 μm.

After injection and incubation for 2 h, some IILs penetrated the enteric mucosa (Figure 11). When the IILs were pre-incubated with rTsSerp, TRX-tag, anti-rTsSerp serum, anti-TRX-tag serum or PBS, the percentages of IILs that invaded the intestinal mucosa were 71.33, 45.67, 55.34, 57.6 and 56.00%, respectively. rTsSerp evidently accelerated larval invasion relative to that in the TRX-tag and PBS groups (*χ*^2^ = 6.000, *P* < 0.0001), whereas relative to anti-TRX-tag serum, anti-rTsSerp serum significantly suppressed worm invasion of the gut epithelium (*χ*^2^ = 4.900, *P* < 0.0001).

### NBL destruction by ADCC

**Figure 12:**
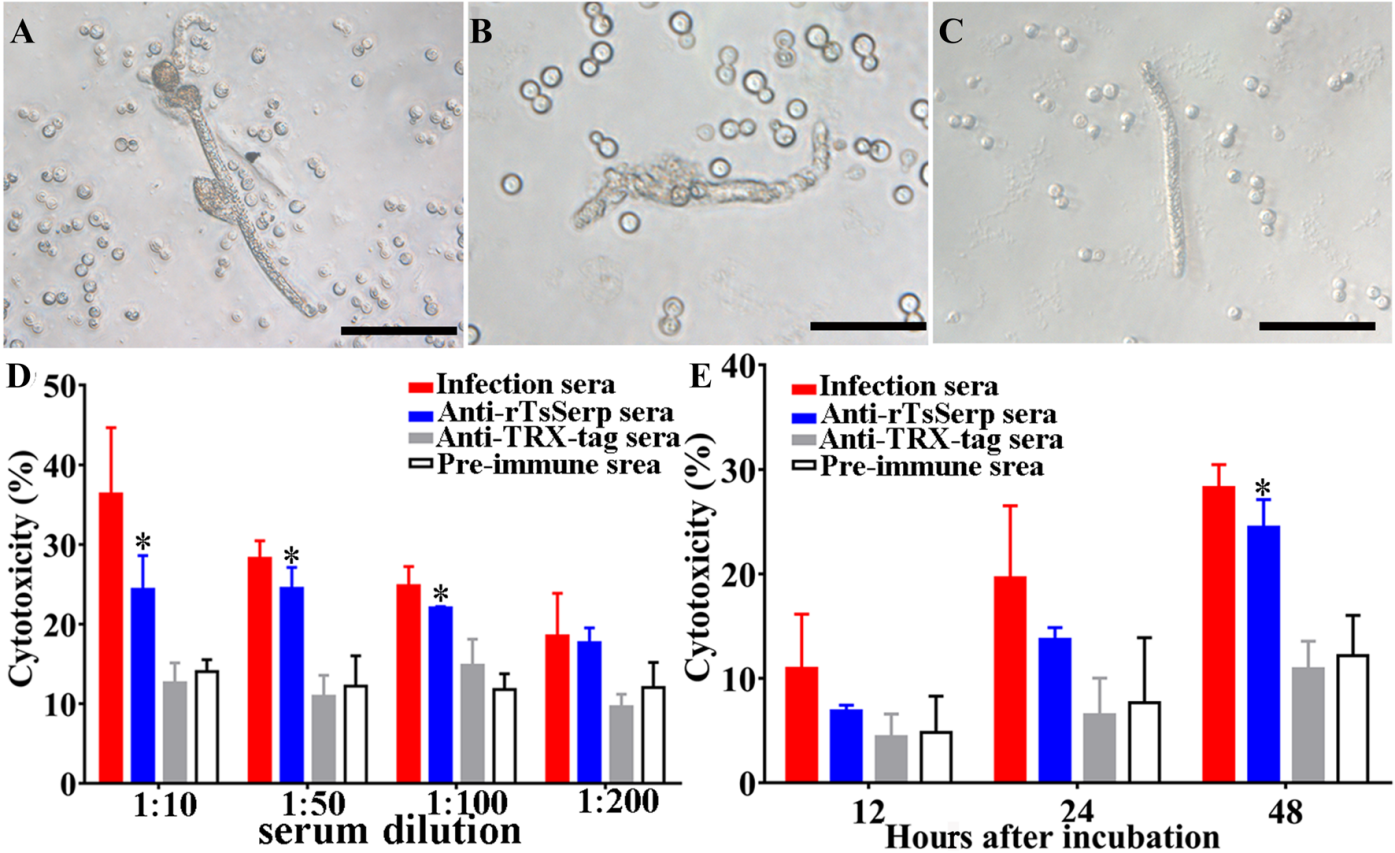
**ADCC killing effect on NBLs.** NBLs were cultured with anti-rTsSerp serum and 2 × 10^5^ mouse peritoneal exudate cells (PECs) at 37 °C for 48 h. **A** PECs adhering to the NBLs; **B** Dead and disintegrated NBLs; **C** Live NBLs; **D** The cytotoxicity was dose dependent for anti-rTsSerp antibodies. **E** Cytotoxicity also increased with prolonged culture time. *P < 0.05 relative to the pre-immune serum group. Scale bars: 50 μm.

The results of the ADCC test revealed that after culture at 37 °C for 48 h, anti-rTsSerp antibodies mediated PEC adhesion to the NBLs and damage to the NBLs (Figure 12). When 1:10–1:100 dilutions of anti-rTsSerp serum were added, the ADCC resulted in evident increased death of the NBLs (24.35, 24.47 and 22.02% cytotoxicity) compared with that of the NBLs cultured with pre-immune serum (16.99, 12.18 and 11.77%) (*t*_1:10_ = 3.959, *P* < 0.05; *t*_1:50_ = 4.562, *P* < 0.05; *t*_1:100_ = 8.981, *P* < 0.01). The cytotoxicity was dose-dependently related to anti-rTsSerp antibody concentrations (*r* = 0.903), and the cytotoxicity had a decreasing trend following the increase in serum dilutions (*F* = 119.518, *P* < 0.0001). There was also a positive correlation between cytotoxicity and culture time (*r* = 0.921), and cytotoxicity showed an increasing trend with prolonged culture time (*F* = 18.356, *P* < 0.01).

### Immune protection produced by rTsSerp vaccination

**Figure 13:**
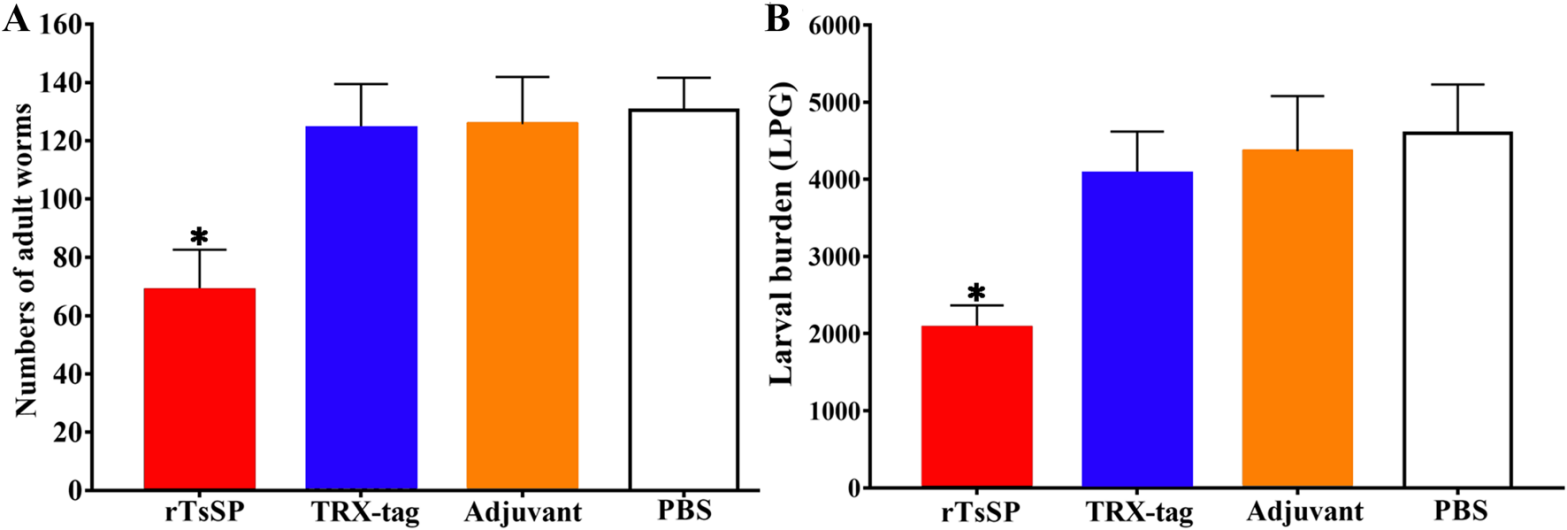
**Immune protection elicited by rTsSerp vaccination following challenge with 300** ***T. spiralis*** **larvae in a murine model. A** Intestinal AW burdens; **B** Muscle larva burden (larvae per gram, LPG). The worm burdens are shown as the mean ± SD from the rTsSerp-vaccinated mouse, TRX-tag, ISA 201 adjuvant and PBS control groups (n = 10). *P < 0.0001 compared to the TRX-tag, ISA 201 adjuvant and PBS groups.

Compared to those in the PBS group, the mice immunized with rTsSerp exhibited a 48.7% reduction in intestinal AWs at 6 dpi (Figure 13A) and a 52.5% reduction in MLs at 56 dpi (Figure 13B) after oral challenge using 300 *T. spiralis* MLs (*F*_AW_ = 120.677, *P* < 0.0001; *F*_ML_ = 131.244, *P* < 0.0001), indicating that vaccination of mice with rTsSerp elicited partial immune protection against *T. spiralis* larval challenge infection.

### Muscle histopathological changes in infected mice

**Figure 14:**
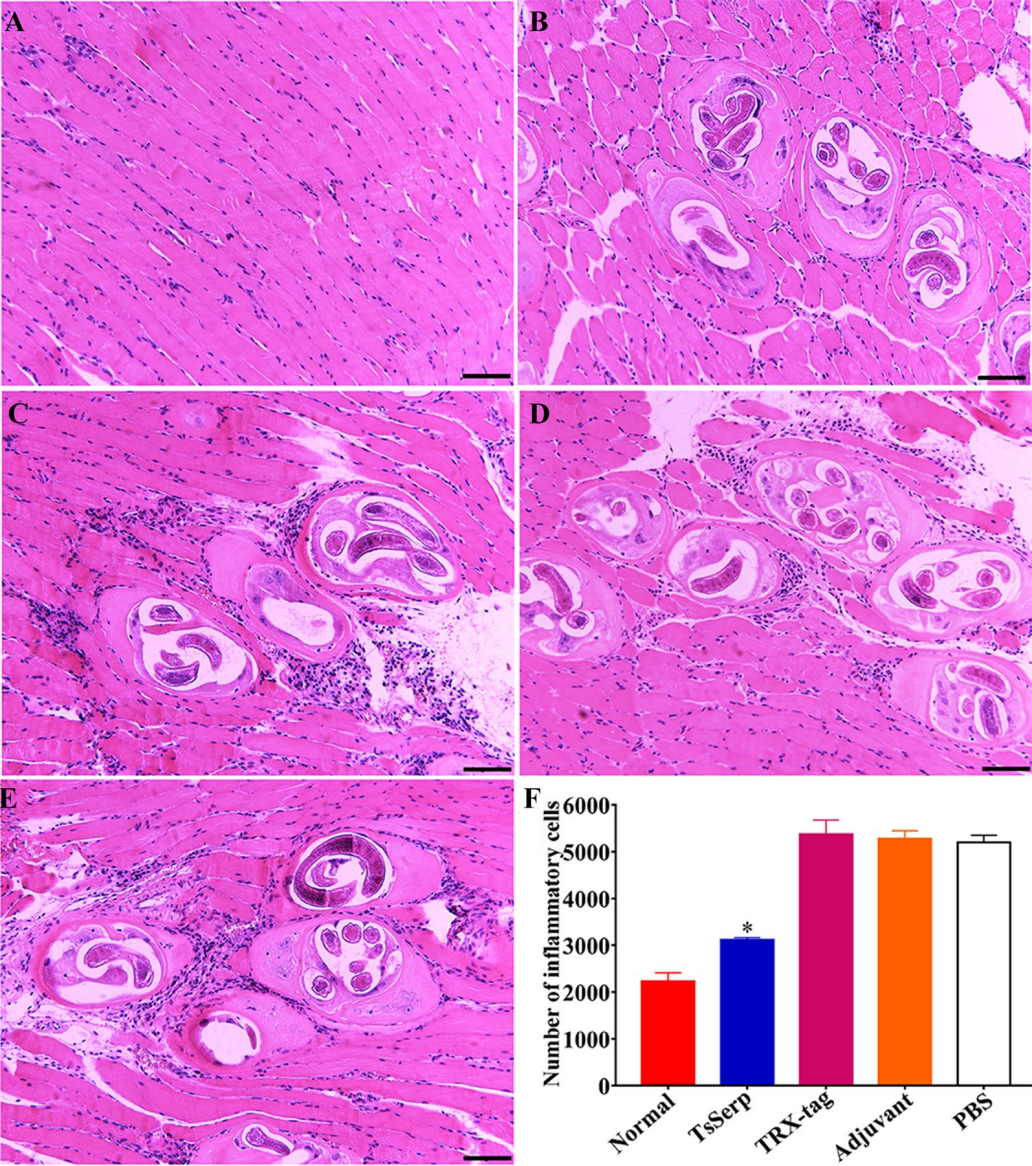
**Muscle histopathological changes in infected mice.** The masseter muscle sections were stained using haematoxylin and eosin (HE) and examined under microscopy. **A** Normal uninfected mice. **B** rTsSerp-vaccinated mice. TRX-tag (**C**), ISA 201 adjuvant (**D**) and PBS (**E**) control mice. **F** Quantification of inflammatory cells around encapsulated *T. spiralis* larvae per field (× 200). *P < 0.0001 compared to the TRX-tag, ISA 201 adjuvant and PBS groups.

The histopathological changes in the masseter muscles from different groups of mice were examined at 56 days after challenge infection with 300 *T. spiralis* MLs. Normal uninfected mice were used as a blank control. As shown in Figure 14, the infiltration of inflammatory cells around encapsulated *T. spiralis* larvae was obviously less than that in the TRX-tag, adjuvant and PBS control groups (*t* = 16.974, *P* < 0.0001), suggesting that rTsSerp immunization alleviated the inflammatory infiltration around the encapsulated larvae.

## Discussion

Serine proteinases are important members of the proteolytic enzyme superfamily, which are widely distributed in organisms. Serine proteases have two main structural folds, trypsin-like domains and subtilisin-like domains. Most trypsin-like domains play an important role in assisting parasites to invade, digest, moult, and hydrolyse proteins [16]. Parasite serine proteases are also involved in reproduction, coagulation, and escape from the host's immune system. Previous studies have shown that *Trichuris muris* serine proteinase can disrupt the integrity of intestinal epithelial cell membranes, which is due to hydrolysis of the mucus barrier of the host intestinal surface [58]. Anti-TspSP-1.2 serum partially prevented the larval invasion of IECs, and the rTspSP-1.2 protein elicited partial immune protection in immunized mice. The silencing of TsSP1.2 by RNAi inhibited larval invasion and development [59]. Mice immunized with another *T. spiralis* serine protease, Ts31, showed a 53.5% reduction in muscle larval burden after larval challenge [60]. These results suggest that the serine proteases might be related to the larval invasion of the enteric mucosa and could be potential targets for anti-*Trichinella* vaccines.

In the present study, TsSerp was cloned into the pET-32a plasmid and expressed in an *E. coli* expression system. Bioinformatics analysis showed that TsSerp has a signal peptide and one functional trypsin-like serine protease domain with an active site containing the classic catalytic triad. Sequence analysis showed that TsSerp has 86.9% identity with a serine protease of *T. nelsoni*. The phylogenetic tree indicated that TsSerp has a close evolutionary relationship with the serine protease of *T. nelsoni* and demonstrated a monophyletic group of 12 species/genotypes of the genus *Trichinella*. Following purification, rTsSerp was immunogenic and used to produce anti-rTsSerp antibodies. Immunization of mice with rTsSerp induced a specific anti-rTsSerp antibody response, and the titre of specific anti-rTsSerp IgG in immune serum reached 1:10^5^. The enzymatic activity of rTsSerp was not observed by using gel zymography or a specific substrate (data not shown). The absence of serine protease enzymatic activity of rTsSerp expressed in this study is likely because of incorrect folding of rTsSerp in a prokaryotic expression system. Therefore, to obtain rTsSerp with enzymatic activity, a eukaryotic expression system is required to express this protein.

Western blotting results showed that rTsSerp was recognized by anti-rTsSerp serum and *Trichinella*-infected mouse serum. RT-PCR showed that TsSerp mRNA expression was observed at diverse *T. spiralis* phases (ML, IIL, 3-day AW and NBL), indicating that the TsSerp gene was transcribed at all *T. spiralis* developmental phases. Western blotting revealed that several native TsSerp protein bands of 18.8–83.7 kDa in ML crude and ES proteins were recognized by anti-rTsSerp serum, likely because TsSerp protein might have various isoforms, this protein might be processed by post-translational processing and modification, or TsSerp is a member of the *Trichinella* serine protease superfamily that possesses the same antigenic epitopes [12, 36, 40]. Western blotting results also demonstrated that TsSerp was a secretory protein, suggesting that TsSerp can be exposed to the host’s enteric local mucosa and elicit the generation of anti-*Trichinella* antibodies [23]; the TsSerp expression levels in MLs and IILs were obviously higher than those in the AW and NBL stages. The results suggested that TsSerp might play an important role in the process of IIL invasion.

The IFA results showed that natural TsSerp was also expressed at various *T. spiralis* lifecycle phases and that it was localized mainly at the cuticle and stichosome of *T. spiralis* MLs and IILs and female intrauterine embryos, suggesting that TsSerp, as a surface protein, also participates in *T. spiralis* invasion and development in the host [37]. *T. spiralis* IIL surface proteins are exposed to and contacted directly by host enterocytes, and they might mediate the larval invasion of the enteric mucosa [15, 17]. In the in vitro larval invasion of enterocytes and excised gut, an evident promotion effect of rTsSerp protein on the larval penetration of IECs and the enteric mucosa was observed, and the promotion was rTsSerp dose dependent and could be due to the specific binding between rTsSerp and IECs [51]. Furthermore, the capacity of *T. spiralis* IILs to penetrate into IECs and the enteric mucosa was prominently suppressed by anti-rTsSerp antibodies, and the suppressive role of the anti-rTsSerp antibodies was dose dependent. The anti-rTsSerp antibody inhibitory effect on larval invasion is likely due to the formation of a cap-like immune complex of TsSerp and anti-TsSerp IgG at the larval anterior, which impedes the direct contact between the nematode and IECs, blocking worm invasion [60]. When *T. spiralis*-infected mouse serum was used in the in vitro invasion test, its inhibitory effect on invasion was more evident than that of anti-rTsSerp serum. This result is likely because the antibodies to other *T. spiralis* invasion-related proteins (e.g., cysteine protease, cathepsin and aminopeptidase, etc.) in infection serum also had an inhibitory effect on epithelial invasion [61]. However, an intestinal loop model might be more suitable than the excised intestine to assess the larval invasion of the intestinal mucosa [62], and it needs to be applied in further study. Furthermore, it is necessary to characterize which enterocyte proteins interact with TsSerp through co-immunoprecipitation and mass spectrometry in future experiments.

To evaluate the protective immunity produced by rTsSerp immunization, the humoral and cellular immune response triggered by rTsSerp vaccination was ascertained in this study. The results showed that vaccination of mice with rTsSerp elicited a remarkable humoral immune response (high levels of serum IgG, IgG1/IgG2a subclasses, IgE and IgM), and it also triggered both systemic (spleen) and local intestinal mucosal (MLN) cellular immune responses, as demonstrated by a significant elevation of Th1 cytokines (IFN-γ) and Th2 cytokines (IL-4) after spleen and MLN cells from vaccinated mice were stimulated with rTsSerp. The mixed Th1/Th2 immune response is crucial for protective immunity against *Trichinella* challenge [63]. Furthermore, IL-2, which is involved in the proliferation of T cells, also plays a significant role in resistance to *Trichinella* infection [22, 64]. Specific anti-*Trichinella* IgG participated in the killing and destruction of newborn larvae through ADCC [55, 56]. To evaluate the cytotoxicity of anti-TsSerp antibodies, an in vitro ADCC test was performed in this study. The results revealed that TsSerp-specific antibodies facilitated macrophage adherence to and killing of the NBLs, and ADCC was dose dependent for anti-TsSerp antibodies. Moreover, the high levels of serum anti-TsSerp IgG and IgE might play an important role in rapid expulsion of the IILs and adult worms from the intestine of vaccinated mice and in delaying the larval invasion of the enteric mucosa following challenge infection [47]. IgE exerts a crucial function in the enteric lumen and is transported from the blood. IgE binds to the *T. spiralis* worm surface and mediates mast cell degranulation to impede larval invasion [65]. Additionally, IgE also plays a vital role in the killing and destruction of NBLs in an ADCC fashion. Our results indicated that vaccination of mice with rTsSerp resulted in a partial reduction in enteric and muscle worm burdens in mice immunized with rTsSerp. The results suggested that the specific antibody response and systemic/enteric mucosal cellular immune response are important for immune protection against *T. spiralis* infection.

Furthermore, the inflammatory infiltration around encapsulated *T. spiralis* larvae in the muscles of immunized mice after challenge infection was obviously less than that in the mice in the three control groups, indicating that immunization with rTsSerp appeared to alleviate inflammatory infiltration of muscle tissues in immunized mice. This effect is likely because the IL-10 produced by rTsSerp immunization limits the inflammatory responses to the developing larvae during the muscle phase of *Trichinella* infection [22, 66].

*T. spiralis* is a multicellular intestine- and tissue-parasitizing parasite with a complex life cycle, and different developmental stages have stage-specific antigens. The immune responses induced by vaccination with an individual *T. spiralis* molecule might not be enough to defend against larval challenge [67]. In this study, subcutaneous vaccination of mice with TsSerp produced only a 52.5% muscle larval reduction in the tissues, and *T. spiralis* larvae were not completely eradicated in vaccinated animal muscles. The level of protection obtained with subcutaneous vaccination of mice with TsSerp protein is lower than that in previous reports using oral or intranasal vaccination [21, 63, 68].

Since *T. spiralis* infection tends to be chronic, the protective immunity elicited by anti-*Trichinella* vaccines should disable, degrade and dislodge the parasites to eliminate parasites from the gut [67]. Because serine proteinases have multiple biological roles during *Trichinella i*nfection, the co-expression of TsSerp with the genes encoding cytokines, chemokines, and other immune costimulatory molecules might improve the immune responses induced in experimental animal models against *T. spiralis* infection. Furthermore, *T. spiralis* infection mainly results from oral ingestion of infected animal meat and meat-derived products. As *Trichinella* IILs first invade the intestinal columnar epithelium and dwell in the gut mucosa, subcutaneous immunization is not an appropriate vaccination route for an anti-*Trichinella* vaccine. Intranasal vaccination of mice with attenuated *Salmonella* expressing a *T. spiralis* gp43 antigen-derived 30-mer peptide fused to the molecular adjuvant C3d-P28 produced a 92.8% reduction in enteric adult worms after larval challenge [63]. When the mice were orally administered recombinant *Lactobacillus plantarum* expressing murine IL-4, the vaccinated mice exhibited an 83.3% reduction in muscle larval burden at 28 dpi [69]. An oral or intranasal vaccination route should be more effective to induce mucosal antibodies and cellular immunity, which are long-lasting and provide substantial protection against the intestinal stages of the parasite [63]. A multiple antigenic epitope vaccine has strong immunogenicity and could enhance the immune response and protective effect in immunized animals [50]. Therefore, an effective preventative vaccine should be composed of multiple *Trichinella* protective antigens (e.g., diverse *T. spiralis* serine proteases and cysteine proteases) that can elicit protective immunity against IILs and adult stages, and oral polyvalent preventive vaccines against diverse *T. spiralis* target antigens of intestinal stage worms should be developed to eliminate the parasite from the gut and thus to prevent the production and migration of NBLs.

To orally vaccinate animals, a recombinant *Trichinella* DNA vaccine needs to be delivered by live carriers (attenuated *Salmonella* or *Lactobacillus*). Lactic acid bacteria (species of the genus *Lactobacillus*) are commonly used probiotics and have obvious advantages for maintaining intestinal homeostasis and enhancing protective immunity [70]. Compared with conventional attenuated *Salmonella* vaccines, a recombinant *Lactobacillus* vaccine is easily prepared, and it is an ideal delivery vector for veterinary vaccines. Probiotics are promising for use in the development of oral polyvalent anti-*Trichinella* vaccines [69]. Additionally, the immune protection obtained in vaccination against *Trichinella* larval challenge in a murine model may not be applicable for other animals. As pork is a main source of human *Trichinella* infection, from a veterinary point of view, the ultimate confirmatory experiment of anti-*Trichinella* vaccines needs to be conducted in a model of domestic pigs.

In conclusion, TsSerp is a secretory protein that is highly expressed at the *T. spiralis* IIL and ML stages and is primarily located at the cuticle, stichosome and intrauterine embryos of the parasite. rTsSerp promoted the larval invasion of IECs and the enteric mucosa, whereas anti-rTsSerp antibodies impeded larval invasion; the promotion and obstruction role was dose-dependently related to rTsSerp or the anti-rTsSerp antibodies, respectively. Vaccination of mice with rTsSerp elicited an evident humoral and cellular immune response that produced partial immune protection against *T. spiralis* larval challenge. These results indicated that TsSerp participates in *T. spiralis* invasion and development in the host and might be considered a potential candidate target antigen to develop oral polyvalent preventive vaccines against *Trichinella* infection.

## Data Availability

Not applicable.

## Abbreviations

ADCC: antibody-dependent cellular cytotoxicity
AWs: adult worms
ES: excretory/secretory
FBS: foetal bovine serum
IECs: intestinal epithelial cells
IFA: immunofluorescence assay
IILs: intestine infective larvae
LPG: larvae per gram
MLs: muscle larvae
MLNs: mesenteric lymph nodes
MW: molecular weight
NBLs: newborn larvae
PBST: PBS-Tween-20
PECs: peritoneal exudate cells
pI: isoelectric point
SD: standard deviation
TBST: tris-buffered saline- 0.5% Tween-20
TsSerp: *T. spiralis* Serine proteinase
